# STAT3 as a potential therapeutic target in triple negative breast cancer: a systematic review

**DOI:** 10.1186/s13046-019-1206-z

**Published:** 2019-05-14

**Authors:** Jiang-Jiang Qin, Li Yan, Jia Zhang, Wei-Dong Zhang

**Affiliations:** 1 College of Pharmaceutical Science, Zhejiang Chinese Medical University, 548 Binwen Road, Binjiang District, Hangzhou, 310053 Zhejiang China; 2 School of Pharmacy, Naval Medical University, 325 Guohe Road, Yangpu District, Shanghai, 200433 China; 3 Shanxi Institute of Traditional Chinese Medicine, Taiyuan, 030012 China; 4 Institute of Interdisciplinary Integrative Medicine Research, Shanghai University of Traditional Chinese Medicine, Shanghai, 201203 China

**Keywords:** STAT3, Triple negative breast cancer, Oncogene, Immune escape, Small molecule inhibitors

## Abstract

Triple negative breast cancer (TNBC), which is typically lack of expression of estrogen receptor (ER), progesterone receptor (PR), and human epidermal growth factor receptor 2 (HER2), represents the most aggressive and mortal subtype of breast cancer. Currently, only a few treatment options are available for TNBC due to the absence of molecular targets, which underscores the need for developing novel therapeutic and preventive approaches for this disease. Recent evidence from clinical trials and preclinical studies has demonstrated a pivotal role of signal transducer and activator of transcription 3 (STAT3) in the initiation, progression, metastasis, and immune evasion of TNBC. STAT3 is overexpressed and constitutively activated in TNBC cells and contributes to cell survival, proliferation, cell cycle progression, anti-apoptosis, migration, invasion, angiogenesis, chemoresistance, immunosuppression, and stem cells self-renewal and differentiation by regulating the expression of its downstream target genes. STAT3 small molecule inhibitors have been developed and shown excellent anticancer activities in in vitro and in vivo models of TNBC. This review discusses the recent advances in the understanding of STAT3, with a focus on STAT3’s oncogenic role in TNBC. The current targeting strategies and representative small molecule inhibitors of STAT3 are highlighted. We also propose potential strategies that can be further examined for developing more specific and effective inhibitors for TNBC prevention and therapy.

## Background

Triple negative breast cancer (TNBC) is the most aggressive form of breast cancer and accounts for much higher recurrence and metastasis rates [1]. Due to the absence of the expression of estrogen receptor (ER), progesterone receptor (PR), and human epidermal growth factor receptor 2 (HER2), TNBC is unresponsive to endocrine and HER2-targeted therapies, which results in the high mortality of patients with this disease [1]. When patients are diagnosed with TNBC at the early stage, combination chemotherapy (anthracyclines, taxanes, platinum salts, etc.) with or without radiotherapy is used as standard non-surgical therapy and has shown some efficacy in patients with both primary and metastatic diseases [2]. Because of the inter- and the intratumoral heterogeneities of TNBC, the intrinsic chemoresistance as well as severe side effects are often observed and lead to limited success in the clinic [3, 4]. Targeted therapies (e.g.*,* poly (ADP-ribose) polymerase (PARP) inhibitors and epidermal growth factor receptor (EGFR) inhibitors) and immunotherapies have also shown some promise in preliminary clinical studies, but further investigations are critically needed [5–7]. More recently, many efforts have been made to identify targetable molecules for treating TNBC via genomic profiling and several critical alternations have been discovered, including the overexpression and aberrant activation of signal transducer and activator of transcription 3 (STAT3) [8, 9]. The emerging data suggest that STAT3 may be a potential molecular target and biomarker for TNBC.

The STAT family of transcription factors is comprised of seven members with high structural and functional similarity, including STAT1, STAT2, STAT3, STAT4, STAT5a, STAT5b, and STAT6 [10, 11]. All STAT proteins consist of an amino acid domain (NH_2_), a coiled-coil domain (CCD) for binding with interactive proteins, a DNA binding domain (DBD), a linker domain, a SRC homology 2 (SH2) domain for phosphorylation and dimerization, and a C-terminal transactivation domain (TAD) [11]. Most of these domains are highly conserved among STAT proteins and only TAD is divergent and mainly contributes to their structure diversity [12]. STAT3 was initially discovered to bind to DNA in response to interleukin-6 (IL-6) and epidermal growth factor (EGF) in 1994 [13, 14]. Over the past decades, STAT3 has become one of the most investigated oncogenic transcription factors and is highly associated with cancer initiation, progression, metastasis, chemoresistance, and immune evasion [15, 16]. The recent evidence from both preclinical and clinical studies have demonstrated that STAT3 plays a critical role in TNBC and STAT3 inhibitors have shown efficacy in inhibiting TNBC tumor growth and metastasis.

Considering that there is an unmet medical need for TNBC treatment and innovative therapeutic agents are urgently required, an in-depth understanding of the roles of STAT3 in TNBC will facilitate the development of STAT3-targeted therapeutics and pave the way for a novel TNBC treatment approach. In this review, we focus on the recent findings related to STAT3’s role in TNBC as well as STAT3 inhibitors and current targeting strategies. We also discuss other potential strategies for developing new STAT3 inhibitors for TNBC treatment.

## The STAT3 signaling pathway

**Fig. 1:**
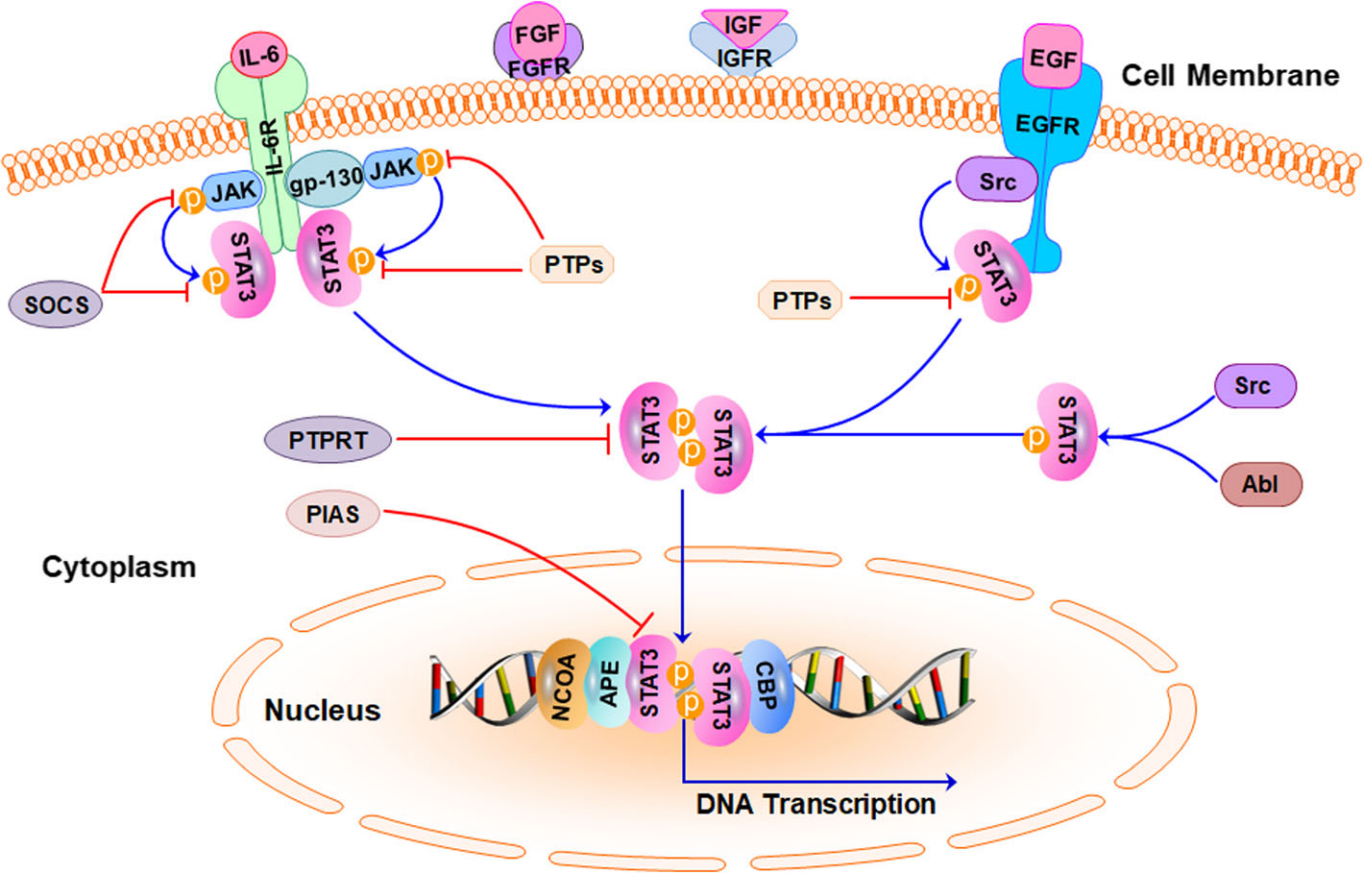
The STAT3 signaling pathway in cancer cells. Under normal physiological conditions, STAT3 activation is strictly controlled by the endogenous inhibitors, including the protein inhibitor of activated STAT (PIAS), the suppressor of cytokine signaling (SOCS), and several protein tyrosine phosphatases (PTPs). Once the upstream cytokines (e.g.*,* IL-6) or growth factors (e.g.*,* EGF, FGF, and VEGF) bind to cell surface receptors, STAT3 is phosphorylated and activated by JAK or Src. The nonreceptor tyrosine kinases (e.g.*,* Src and Abl) also phosphorylate STAT3. The phosphorylated STAT3 undergoes dimerization and translocates from cytoplasm into the nucleus. The activated STAT3 further binds to DNA and its coactivators (e.g.*,* NCOA, APE, and CBP) and induces the transcription of its downstream target genes

The classical STAT3 signaling pathway that is activated through the binding of cytokines or growth factors to their corresponding cell surface receptors has been extensively reviewed [16–18]. Here, we present a brief overview of the STAT3 signaling pathway, nonreceptor tyrosine kinases of STAT3, and its intrinsic inhibitors and coactivators, which are depicted in Fig. 1. Briefly, the overexpressed cytokine receptors, e.g., interleukin-6 receptor (IL-6R) and interleukin-10 receptor (IL-10R) and the hyperactive growth factor receptors, e.g., epidermal growth factor receptor (EGFR), fibroblast growth factor receptor (FGFR) and insulin-like growth factor receptor (IGFR) always trigger the tyrosine phosphorylation cascade through the binding of ligands to these receptors, leading to the aberrant activation of STAT3 and the transcription of its downstream target genes [17]. Once the ligands bind to their receptors on the cell surface, these receptors further form dimers and successively recruit glycoprotein 130 (gp130) and Janus kinases (JAKs), thus phosphorylating and activating JAKs [19]. Conversely, the cytoplasmic tyrosine residues of these receptors are phosphorylated by the activated JAKs and then interact with the SH2 domain of STAT3, resulting in STAT3 phosphorylation at Tyr705 by JAKs [16]. In addition, STAT3 can be phosphorylated and activated by several nonreceptor tyrosine kinases, e.g.*,* Src and Abl [20]. The phosphorylated STAT3 (pSTAT3) further forms a homodimer through interaction between their phosphorylated Tyr705 site and SH2 domain, triggering the dissociation of STAT3 dimers from the cell surface receptors and its translocation from cytoplasm to the nucleus [21, 22]. With the help of a variety of coactivator proteins, including NCOA/SRC1a, apurinic/apyrimidinic endonuclease-1/redox factor-1 (APE/Ref-1), and CREB-binding protein (CBP)/p300, the nuclear STAT3 binds to specific DNA sequences and activates the transcription of genes that regulate various phenotypes of cancer cells [17, 18].

STAT3 is also highly expressed in some normal tissues and organs, including the bone marrow, peripheral nervous system, and digestive tract and plays a physiological role [23–25]. In the normal physiological conditions, STAT3 phosphorylation and activation are tightly controlled by several intrinsic inhibitors, including protein tyrosine phosphatases (PTPs), the suppressors of cytokine signaling (SOCS), and the protein inhibitor of activated STAT (PIAS) [26]. The Src homology domain-containing tyrosine phosphatases 1/2 (SHP-1/2) directly interact and dephosphorylate JAK and STAT3, resulting in their inactivation [27, 28]. The nuclear PTPs, including TC45 and T-cell protein-tyrosine phosphatase (TC-PTP) induce the inactivation of STAT3 through its dephosphorylation and translocation from nucleus to the cytoplasm [29, 30]. Other PTPs, such as PTP1B and PTPeC have also been reported to regulate STAT3 dephosphorylation and inactivation [31]. Moreover, SOCS directly interacts with JAK and STAT3 and inhibits their phosphorylation and activation via forming a negative feedback loop with JAK-STAT3 signaling pathway [32]. PIAS inhibits the binding of nuclear STAT3 to DNA and induces STAT3 dephosphorylation via protein tyrosine phosphatase receptor T (PTPRT), leading to the reduced expression of its downstream target genes [33]. In addition, the stability of STAT3 protein is also regulated by the ubiquitin-proteasome system via the ubiquitin ligase TRAF6 (tumor necrosis factor receptor-associated factor 6) [34]. Recent studies have also reported that miR-544 directly targets the 3′-untranslated region (UTR) on *STAT3* mRNA, thus down-regulating STAT3 expression in TNBC cells [35]. Due to the presence of these endogenous inhibitors, STAT3 is strictly governed to exert its physiological functions in normal cells [36]. Herein, both direct inhibition of STAT3 and activation of the endogenous inhibitors may be considered as potential STAT3-inhibiting strategies for developing novel cancer therapeutics.

## The STAT3 signaling pathway in triple negative breast cancer

**Fig. 2:**
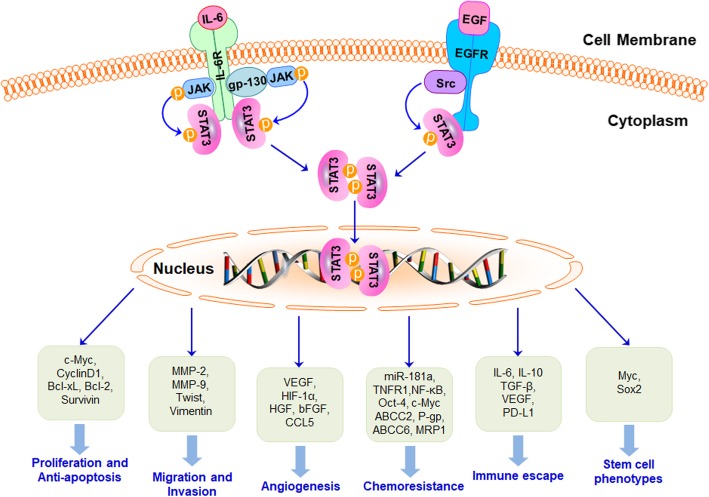
Activation of STAT3 signaling promotes growth, metastasis, chemoresistance, immune escape, and stemness in TNBC. One the upstream regulators are activated, STAT3 is phosphorylated, dimerized, and translocated into the nucleus, where it activates the transcription of the target genes that regulate cell proliferation, anti-apoptosis, migration, invasion, angiogenesis, chemoresistance, immune escape, stem cell phenotypes, and autophagy

The oncogenic potential of STAT3 has been widely recognized through its involvement in regulating the expression of genes related to cancer cell proliferation, anti-apoptosis, migration, invasion, angiogenesis, chemoresistance, immune suppression, stem cell self-renewal and maintenance, and autophagy (as shown in Fig. 2) [17, 18]. Importantly, STAT3 is overexpressed and constitutively activated in TNBC, which is highly related to TNBC initiation, progression, metastasis, resistance to chemotherapy, and the poor survival outcomes [8]. STAT3 is not only capable of eliciting the expression of cancer-related genes, but also physically interacts and functionally cooperates with other oncogenic transcription factors, e.g., GLI1, promoting the aggressiveness of TNBC [8]. A recent study has also found a reduction of the gene associated with retinoic-interferon-induced mortality 19 (GRIM-19), an intrinsic inhibitor of STAT3 transcription accompanied by STAT3 overexpression in TNBC [37]. In addition, TCPTP, including two splice variants TC45 and TC48 are down-regulated in TNBC cells in vitro and in vivo, which also contributes to the activation of STAT3 signaling [38]. Indeed, STAT3 has also been found to localize in the mitochondria, where it is termed mitoSTAT3 and regulates the mitochondrial functions, including electron transport chain, ATP synthesis, calcium homeostasis, and reactive oxygen species (ROS) accumulation [39, 40]. Moreover, mitoSTAT3 has been shown to promote breast cancer cell growth, in which the phosphorylation of Serine 727 plays a critical role [41].

A recent study has shown that acetylated STAT3 is highly elevated in TNBC, causing the methylation and inactivation of tumor-suppressor gene promoters [42]. Importantly, mutation of STAT3 at Lys685 or reducing STAT3 acetylation by resveratrol could induce demethylation and activation of the estrogen receptor-α gene and sensitize TNBC cells to antiestrogens. Considering the emerging data that demonstrate the critical role of STAT3 in TNBC, we herein present a comprehensive overview of its oncogenic functions in this section.

### Role of STAT3 in TNBC cell proliferation and anti-apoptosis

Several studies have demonstrated that STAT3 promotes cell proliferation and inhibits apoptosis in TNBC by increasing the expression of target genes, including survivin, c-Myc, cyclin D1, B-cell lymphoma-2 (Bcl-2), and B-cell lymphoma-extra large (Bcl-xL) [21]. In TNBC, STAT3 directly binds to the *survivin* promoter and promotes its transcription [43, 44], which can be blocked by inhibiting the nuclear export factor, exportin 1 (XPO1) and CBP-mediated STAT3 acetylation [45]. In addition, Galectin-1, a β-galactoside binding protein has also been shown to contribute to TNBC progression through binding to integrin β1 and activating the integrin β1/FAK/c-Src/ERK/STAT3/survivin pathway [46]. Conversely, WW domain-containing oxidoreductase (Wwox) inhibits TNBC cell proliferation by interacting with JAK2 and suppressing JAK2 and STAT3 phosphorylation [47]. Wwox also represses the binding of STAT3 to the *IL-6* promoter, therefore decreasing the expression of IL-6 cytokine. A tumor suppressor gene, gametogenetin-binding protein 2 (GGNBP2) has been found to inhibit breast cancer cell proliferation and induce apoptosis, independent of ER expression [48]. A further study has indicated that the inhibition of IL-6/STAT3 signaling by GGNBP2 is mainly responsible for its inhibitory effects on TNBC growth and metastasis [48].

STAT3 also promotes TNBC cell proliferation and inhibits apoptosis through the crosstalk with SET and MYND domain 2 (SMYD2) and nuclear factor-kappa B (NF-κB) [49]. SMYD2 is highly expressed in TNBC cell lines and tissues, which is correlated with increased TNBC cell proliferation and survival. Mechanistically, SMYD2 physically interacts with STAT3 and NF-κB p65 and increases their methylation and phosphorylation, promoting tumor growth and metastasis [49]. STAT3 recruits the acetyltransferase p300 to enhance NF-κB acetylation and prolong its nuclear retention [50]. In addition, STAT3 and NF-κB also contribute to each other’s activation via SMYD2 [49]. Interestingly, a recent study has reported an opposite role of STAT3 in TNBC cells [51]. It was observed that STAT3 knockdown did not inhibit but promoted the growth of MDA-MB-231 cells-derived xenograft tumors, implying that the oncogenic role of STAT3 in TNBC might be context-specific [51].

### Role of STAT3 in TNBC cell migration and invasion

The role of STAT3 in promoting cell migration and invasion has been linked to the upregulated expression of matrix metalloproteinase 2 (MMP2), MMP9, TWIST, and Vimentin [52]. As discussed earlier, the STAT3 signaling is frequently activated through the binding of cytokines and growth factors to their corresponding receptors in cancer cells. A newly discovered cytokine termed interleukin-22 (IL-22) was recently reported to promote the migration of TNBC cells and induce their chemoresistance by activating the JAK/STAT3/MAPKs/AKT signaling pathway. The increased levels of the IL-22 producing (Th22) cells were also observed in normal, paratumor, and tumor tissues from patients with TNBC, which confirmed the importance of IL-22/JAK/STAT3/MAPKs/AKT in metastasis of this disease [53].

Recent studies reported that several upstream regulators of STAT3 signaling are involved in TNBC metastasis. Wwox blocks JAK2-STAT3 interaction and inhibits STAT3 phosphorylation, therefore repressing STAT3-driven TNBC metastasis [47]. G protein-coupled estrogen receptor (GPER) has been demonstrated as a TNBC metastasis suppressor. Mechanistically, activation of GPER can inhibit the NF-κB/IL-6/STAT3 signals, cause STAT3 dephosphorylation and inactivation, and then suppress migration and angiogenesis of TNBC [54]. GPER also triggers Y397 phosphorylation of focal adhesion kinase (FAK) in TNBC while the activation of both GPER and FAK promotes the migration of TNBC cells by increasing STAT3 nuclear accumulation and gene expression [55].

### Role of STAT3 in angiogenesis of TNBC

The pro-angiogenic role of STAT3 has been partially attributed to the upregulation of vascular endothelial growth factor (VEGF), hypoxia-inducible factor 1-alpha (HIF-1α), hepatocyte growth factor (HGF), and basic fibroblast growth factor (bFGF) via STAT3 transactivation [52]. A recent study demonstrated that lymphatic endothelial cells (LECs) promote angiogenesis and metastasis through pSTAT3-mediated CCL5 expression in TNBC [56]. LECs are an important component of lymphatic vessels (LVs), which are prevailingly considered as the routes for cancer metastasis. Lee et al. have found that IL-6 secretion from TNBC cells causes STAT3 phosphorylation and activation, therefore inducing HIF-1α and VEGF expression. pSTAT3 also forms a ternary complex with phosphorylated c-Jun (pc-Jun) and phosphorylated activating transcription factor 2 (pATF2), which induces CCL5 expression in LECs and accelerates metastasis [56]. It was also observed that estrogen activates G protein-coupled estrogen receptor-1 (GPER-1), inhibits the expression VEGF at both protein and mRNA levels, and suppresses the tumor growth and angiogenesis in TNBC xenograft tumor models, in which STAT3 is involved [57].

### Role of STAT3 in chemoresistance of TNBC

It has frequently been observed that blocking STAT3 signaling enhances the anticancer activity of chemotherapies in TNBC cells in vitro and in vivo, which endorses a critical role of STAT3 in chemosensitivity of TNBC [58–61]. Several recent studies revealed the mechanisms underlying STAT3-mediated chemoresistance in different subsets of TNBC cell lines [62]. NF-κB is highly associated with resistance to cancer therapies, while the overexpression and constitutive activation STAT3-NF-κB signaling pathway have been shown to confer chemoresistance in TNBC cells [63]. Mechanistically, STAT3 upregulates the expression of a target gene TNFRSF1A (tumor necrosis factor receptor superfamily member 1A), which recruits TNFα to the cell surface and triggers the activation of NF-κB signaling pathway [64]. The aberrant activation of STAT3 also increases the expression levels of pluripotency transcription factors octamer-binding transcription factor-4 (Oct-4) and c-Myc, which regulate stemness-mediated doxorubicin resistance in TNBC [65]. The restoration of doxorubicin sensitivity of TNBC cells by a STAT3 inhibitor WP1066 further confirms a pivotal role of this oncogene in chemoresistance.

STAT3-mediated microRNA (miRNA) expression is emerging as a mechanism for regulating chemoresistance in TNBC. Niu et al. found that miR-181a expression is increased in TNBC due to doxorubicin treatment and contributes to acquired resistance and metastasis of this disease through repressing the expression of its target gene Bax (Bcl-2-associated x protein) [66]. Further studies have indicated that pSTAT3 at S727 not only directly binds to *MIR181A1* promoter but also recruits MSK1 (mitogen- and stress-activated protein kinase-1) and stabilizes its binding to *MIR181A1* promoter, facilitating the transactivation [67]. The effectiveness of targeting STAT3-mediated *MIR181A1* transactivation for sensitizing cells to chemotherapy and preventing metastasis has also been validated in a TNBC orthotopic model.

STAT3 is also involved in hypoxia-induced chemoresistance in TNBC [67]. Under hypoxia, the intracellular uptake of chemotherapy, especially cisplatin is dramatically reduced due to the upregulated expression of ATP-binding cassette (ABC) drug transporters. Although the expression level and activity of HIF-1α was increased by hypoxia in TNBC, no significant improvement in chemoresistance was observed in TNBC cells that were treated by HIF-1α siRNA. Intriguingly, STAT3 was found to increase the expression levels of ABC transporters, especially ABCC2 (also known as multidrug resistance protein 2, MRP2) and ABCC6 (also known as MRP6) in hypoxia-treated TNBC cells, therefore conferring chemoresistance to cisplatin [67, 68]. However, another study reported that IL-6-mediated STAT3 activation induces HIF-1α expression in TNBC cells, which consequently attenuates chemotherapy-induced cytotoxicity and cell apoptosis through regulating the expression of apoptosis-related proteins (Bax and Bcl-2) and drug transporters (P-glycoprotein and MRP1) [68]. The transfer RNA-derived fragments (tDRs), particularly tDR-0009 and tDR-7336 are upregulated in TNBC under hypoxia and facilitate the doxorubicin resistance through phosphorylating and activating STAT3 [69]. In addition, the combination treatment with HIF-1α and STAT3 inhibitors significantly enhances the cytotoxicity of cisplatin against TNBC cells and overcomes hypoxia-induced chemoresistance [70]. However, the role of STAT3-induced HIF-1α expression in hypoxia-induced chemoresistance is not clear so far, and further investigation is critically needed.

### Role of STAT3 in immune suppression

Recent findings have established STAT3 as a powerful regulator of tumor-mediated immune suppression [21, 71]. STAT3 is not only overexpressed and activated in cancer cells but also in tumor-associated immune cells, inducing the expression of immune-suppression related genes, including IL-6, IL-10, TGF-β and VEGF and driving the escape of cancer cells from immune-mediated elimination [71]. In TNBC, STAT3 and its homolog STAT1 are also involved in regulating the expression of programmed death ligand 1 (PD-L1), a critical immune checkpoint that modulates the magnitude and the functional profile of T cell responses [72]. PD-L1 and PD-L2 are actually also amplified and overexpressed in TNBC cell lines due to JAK-mediated STAT3 phosphorylation and activation [73]. The mechanism studies have shown that pSTAT1 and pSTAT3 form heterodimers in the cytoplasm and translocate into the nucleus, where the pSTAT1-pSTAT3 dimers bind to the *PD-L1* promoter and activate its transcription [72]. Another study has shown that syntenin1 is highly expressed in TNBC tissues and increases the expression level of PD-L1 by activating STAT3, consequently attenuates the response of TNBC to anti-PD-L1 treatment [74]. Moreover, direct inhibition of STAT3 overcomes the resistance of TNBC to immunotherapies, which confirms its immunosuppressive activity [72, 74].

### Role of STAT3 in TNBC stem cell phenotypes

Early studies on STAT3 signaling disclosed an important role in stem cells self-renewal and differentiation [75]. The increasing evidence has also demonstrated that the constitutive activation of IL-6/STAT3 signaling pathway contributes to the stemness of TNBC stem cells under both normal and hypoxia conditions [76, 77]. In addition, the VEGF-VEGFR-2 binding-induced STAT3 phosphorylation and activation was found to promote the self-renewal of breast cancer cells, especially TNBC cells by upregulating the expression of Myc and Sox2 (SRY-related HMG-box 2) [78]. The crosstalk of STAT3 with NF-κB and Wnt signaling pathways was also observed in TNBC cells and serves as a feed-forward loop for regulating the TNBC stem cell function [79]. Moreover, Syndecan-1 (CD138) is highly expressed in TNBC, especially inflammatory TNBC and contributes to the poor prognosis of this disease [80]. Syndecan-1 was recently reported to promote TNBC stem cells through modulating the STAT3, NF-κB, and Wnt signaling pathways together [76]. Another study by Ibrahim et al. has demonstrated the importance of IL-6/STAT3 signaling pathway in Syndecan-1-modulated cancer stem cell phenotype [81]. Furthermore, Notch and EGFR signaling pathways are also implicated in the modulatory effects of Syndecan-1 on TNBC stem cells [81].

Except for cytokines and growth factors, adipokines, e.g.*,* Leptin are also involved in the constitutive activation of the STAT3 signaling pathway. Leptin and its long form of leptin receptor (LEPRb) are enriched in breast cancer tissues and promote cell proliferation, migration, and angiogenesis [82]. Recently studies have shown that the binding of Leptin to LEPRb initiates the activation of JAK2/STAT3 signaling pathway, which further induces self-renewal and maintains the stem-cell state in TNBC stem cells [83]. Moreover, a new upstream regulator of the LEPR-STAT3 signaling pathway termed hematological and neurological expressed 1-like (HN1L) was also discovered to promote TNBC stem cell properties [84]. HN1L is overexpressed in TNBC tissues and correlates with the shorter survival of patients with this disease. The HN1L silencing experiments further confirmed its regulatory effects on LEPR-STAT3 signaling pathway and on TNBC stem cell population and lung metastasis [84].

### Role of STAT3 in autophagy of TNBC cells

Autophagy is capable of regulating STAT3 phosphorylation status in TNBC cells [85]. Maycotte et al. discovered that the autophagy-dependent survival under unstressed conditions is enriched in TNBC, which reduces the response of cancer cells to therapy. Further studies have indicated that autophagy promotes TNBC cell survival by regulating STAT3 phosphorylation and activation [85]. Therefore, pharmacological inhibition of STAT3 may be a promising strategy for treating autophagy-dependent TNBC.

## Targeting STAT3 for TNBC prevention and therapy

**Fig. 3:**
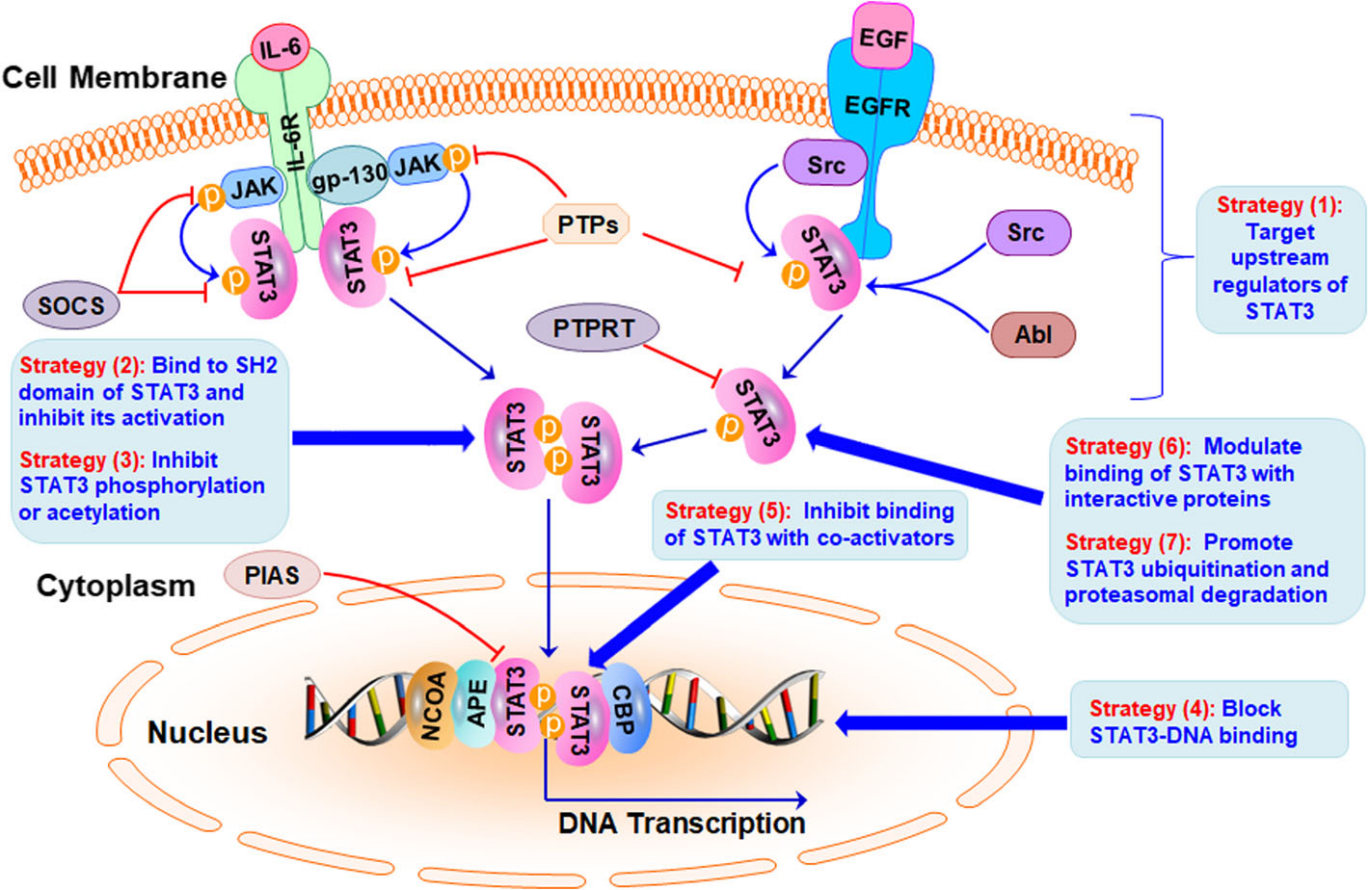
Inhibiting STAT3 signaling at multiple levels for cancer therapy. Currently, the majority of STAT3 inhibitors have been developed through (1) targeting the upstream regulators of STAT3, (2) binding to the SH2 domain of STAT3 and inhibiting its activation, (3) inhibiting STAT3 phosphorylation or acetylation, or (4) blocking STAT3-DNA binding. Other potential strategies, such as (5) inhibiting the binding of STAT3 with its co-activators, (6) modulating the binding of STAT3 with other interactive proteins, and (7) promoting STAT3 ubiquitination and proteasomal degradation may also be evaluated for developing novel STAT3 inhibitors

Abundant evidence has suggested that STAT3 may be a promising molecular target for TNBC therapy [86]. Various STAT3 inhibitors have been developed and shown some efficacy in TNBC models in vitro and in vivo, which have been summarized in Table 1. In this section, we discuss the current STAT3-targeting strategies (as shown in Fig. 3) for treating and preventing TNBC, as well as the challenges in developing more specific and effective STAT3 inhibitors.

**Table 1:** Summary of STAT3 inhibitors and their mechanisms of action for TNBC therapy

Inhibitors	Mechanisms of action	In vitro activity	In vivo activity	Reference
*Strategy 1: Target upstream regulators of STAT3*				
Carfilzomib	Inhibits IL-6/STAT3 signaling pathway	Inhibits mitosis and proliferation and induces apoptosis	Reduces serum IL-6 levels in tumor-bearing mice	[87]
Manuka honey	Inhibits IL-6/STAT3 signaling pathway	Inhibits cell viability and colony formation, induces apoptosis, impairs cell migration and invasion, and inhibits angiogenesis	NR	[88]
Bazedoxifene	Inhibits IL6/gp130/STAT3 signaling pathway	Inhibits cell viability, colony formation and cell migration and synergistically enhances the activity of paclitaxel	Suppresses tumor growth	[89, 90]
*Ganoderma lucidum* extract	Inhibits IL-6/JAK/STAT3 signaling pathway	Inhibits cell viability and induces apoptosis	Suppresses tumor growth	[91]
Arsenic trioxide	Inhibits EZH2/NF-κB/IL-6/STAT3/VEGF signaling pathway	Inhibits angiogenesis	NR	[96]
Deguelin	Inhibits EGFR/STAT3 signaling pathway	Inhibits cell viability	Suppresses tumor growth	[92]
Picrasidine G	Inhibits EGFR/STAT3 signaling pathway	Inhibits cell viability and induces apoptosis	NR	[93]
Cantharidin	Inhibits EGFR/STAT3 signaling pathway	Inhibits cell viability and induces apoptosis	NR	[94]
Silibinin	Inhibits JAK2/STAT3/MMP2 signaling pathway	Inhibits cell viability, migration and invasion	NR	[97]
	Inhibits EGFR/STAT3/Fibronectin signaling pathway	NR	NR	[95]
Ganoderic acid A	Inhibits JAK2/STAT3 signaling pathway	Inhibits cell viability and invasive capacity and induces apoptosis	NR	[98]
Nintedanib	Modulates SHP-1/p-STAT3 signaling pathway	Inhibits cell viability and induces apoptosis	Suppresses tumor growth	[99]
SC-78	Modulates SHP-1/p-STAT3/VEGF-A signaling pathway	Inhibits cell migration and tube formation	Suppresses tumor growth and metastasis	[100]
1,2,3,4,6-penta-O-galloyl-beta-D-glucose	Modulates SHP-1/p-STAT3 signaling pathway	NR	Suppresses tumor growth and metastasis	[101]
SC-2001	Modulates RFX-1/SHP-1/p-STAT3 signaling pathway	Inhibits cell growth and induces apoptosis	Suppresses tumor growth	[95, 102]
Isolinderalactone	Enhances SOCS3-mediated STAT3 dephosphorylation	Inhibits cell viability and colony formation and induces apoptosis	Suppresses tumor growth	[103]
Compound 57	Binds to HSP90 and inhibits the expression and phosphorylation of STAT3	Inhibits cell viability	NR	[104]
L80	Binds to HSP90 and inhibits the expression and phosphorylation of STAT3	Inhibits cell viability induces apoptosis, and suppresses BCSC-like properties	Suppresses the growth of BCSC-enriched TNBC tumors and distant metastasis	[105]
Nor-wogonin	Inhibits TAK1-mediated STAT3 activation	Inhibits cell viability and proliferation and induces G1 and G2/M phases arrest and apoptosis	NR	[106]
Thioridazine	Inhibits DRD2-mediated STAT3 activation	Inhibits cell self-renewal, proliferation, and viability and induces G1 arrest	NR	[107]
*Strategy 2: Directly bind to STAT3 and inhibit its activation*				
Bt354	Directly binds to SH2 domain of STAT3 and inhibits its phosphorylation	Inhibits cell viability, induces G2/M phase arrest and apoptosis, and impairs cell migration	Suppresses tumor growth	[108]
Osthole	Directly binds to STAT3 and inhibits its phosphorylation	Inhibits cell viability and induces G2/M phase arrest and apoptosis	Suppresses tumor growth	[109]
Arctigenin	Directly binds to SH2 domain of STAT3 and inhibits its phosphorylation and DNA binding ability	Inhibits cell viability, induces apoptosis, impairs cell migration and invasion, and sensitizes cells to chemotherapy	Suppresses tumor growth	[110]
Alantolactone	Directly binds to SH2 domain of STAT3 and inhibits its phosphorylation	Inhibits cell viability and colony formation and impairs cell migration and invasion	Suppresses tumor growth	[111]
KYZ3	Directly binds to SH2 domain of STAT3 and inhibits its phosphorylation	Inhibits cell viability, induces apoptosis, and impairs cell migration	Suppresses tumor growth	[113]
*Strategy 3: Inhibit STAT3 phosphorylation or acetylation*				
Sesquiterpene lactones fraction of *Inula helenium* L.	Inhibits STAT3 phosphorylation and nuclear translocation	Inhibits cell viability and induces apoptosis	Suppresses tumor growth	[114]
*Rhus coriaria*	Inhibits STAT3 phosphorylation	Inhibits angiogenesis and impairs cell migration and invasion	Suppresses tumor growth and metastasis	[115]
Schisandrin B	Inhibits STAT3 phosphorylation and nuclear translocation	Inhibits cell viability and colony formation, induces cell cycle arrest and apoptosis, and impairs cell migration	Suppresses tumor growth	[116]
Eupalinolide J	Inhibits STAT3 phosphorylation and activation	Inhibits cell viability	NR	[117]
Galiellalactone analogues 16 and 17	Inhibits STAT3 phosphorylation and activation	Inhibits cell viability	NR	[118]
FZU-03,010	Inhibits STAT3 phosphorylation and activation	Inhibits cell viability and induces G1 phase arrest and apoptosis	NR	[119]
Niclosamide	Inhibits STAT3 phosphorylation and nuclear translocation	Reverses acquired radioresistance	Sensitizes tumors to irradiation	[120]
Flubendazole	Inhibits STAT3 phosphorylation	Inhibits cell viability, induces G2/M phase arrest and apoptosis, and suppresses BCSC-like phenotype	Suppresses tumor growth, angiogenesis and metastasis	[121]
Disulfiram	Inhibits STAT3 expression and phosphorylation	Inhibits cell viability, induces apoptosis, and impairs cancer stem cell-like properties	Suppresses tumor growth and BCSC-like properties	[122]
Salinomycin	Inhibits STAT3 phosphorylation and activation	Inhibits cell viability, promotes anoikis, impairs cell migration and invasion, and decreases CD44^+^/CD24^−^ stem-like population	NR	[123]
Metformin	Inhibits STAT3 phosphorylation	Inhibits cell viability	NR	[124]
SH-I-14	Inhibits STAT3 acetylation and disrupts DNMT1-STAT3 interaction	Inhibits cell viability	Suppresses tumor growth	[126]
*Strategy 4: Block STAT3-DNA binding*				
Methylsulfonyl-methane	Inhibits the bindings of STAT3 to *VEGF* promoter and STAT5 to *IGF-1R* promoter	Inhibits cell viability and induces apoptosis	Suppresses tumor growth	[127]
Isoharringtonine	Inhibits STAT3-mediated Nanog expression	Inhibits cell viability, impairs cell migration, and decreases proportion of BCSC population	NR	[128]
Salidroside	Inhibits the bindings of STAT3 to *MMP2* promoter	Inhibits cell migration, invasion and angiogenesis	NR	[129]

NR, not reported

### Target upstream regulators of STAT3

The majority of STAT3 inhibitors have been identified to target the upstream regulators of STAT3 signaling. STAT3 activation is often initiated through the binding of cytokines and growth factors to their corresponding cell surface receptors. Therefore, small molecules and natural products that are able to inhibit IL-6 secretion and production, e.g.*,* carfilzomib [87], manuka honey [88], bazedoxifene [89, 90], and *Ganoderma lucidum* extract [91] or suppress EGFR expression and phosphorylation, e.g.*,* deguelin [92], picrasidine G [93], cantharidin [94], and silibinin [95] have shown significant inhibitory effects on STAT3 signaling as well as the expression of its downstream target genes in TNBC cell lines. In addition, arsenic trioxide (ATO) was reported to inhibit IL-6-mediated STAT3 activation, consequently reducing the expression of VEGF and suppressing angiogenesis [96]. Further studies have demonstrated that ATO blocks the interaction between enhancer of zeste homolog 2 (EZH2) and NF-κB p65, herein suppressing the activity of NF-κB and reducing the expression of IL-6. All these indirect STAT3 inhibitors have exhibited potent in vitro and in vivo anti-TNBC activities (Table 1). However, most of them have also been found to inhibit other signaling pathways that are triggered by ligand-cell surface receptor binding in cancer cells, indicating a low level of specificity in targeting the STAT3 signaling pathway.

As discussed earlier, several protein tyrosine kinases, such as JAK2 contribute to STAT3 phosphorylation and activation in both receptor-dependent and/or receptor-independent manners. JAK2 inhibitors, including silibinin [97] and ganoderic acid A [98] were found to inhibit TNBC cell viability, migration, and invasion and induce apoptosis in vitro through inhibiting the JAK2/STAT3 signaling pathway. However, their in vivo efficacy still needs further investigation. Targeting the intrinsic STAT3 inhibitors, such as PTPs and SOCS have been considered as a potential strategy for repressing STAT3 signaling pathway. Several natural and synthetic compounds were identified to activate one of the STAT3 PTPs, SHP-1. Among them, nintedanib and SC-78 significantly increase SHP-1 activity without affecting its expression [99, 100], while 1,2,3,4,6-penta-O-galloyl-beta-D-glucose (PGG) and SC-2001 largely induce the expression of SHP-1 [101, 102]. All these SHP-1 activators were also shown to inhibit STAT3 phosphorylation and the expression of its downstream target genes, thus suppressing TNBC cell growth and migration and inducing apoptosis in vitro and in vivo [99–102]. In addition, isolinderalactone was reported to increase SOCS3 expression and then enhance SOCS3-mediated STAT3 dephosphorylation and inactivation [103].

As one of the major client proteins of heat shock protein 90 (HSP90), STAT3 can be degraded through inhibiting HSP90. Two deguelin-derived HSP90 inhibitors, termed compound 57 and L80 have been observed to inhibit STAT3 expression and phosphorylation by interacting with the C-terminal ATP-binding pocket of HSP90 and blocking its function [104, 105]. Both compounds have also exerted their anticancer activities in TNBC models in vitro and in vivo [104, 105]. Moreover, nor-wogonin was found to inhibit the expression of transforming growth factor β-activated kinase 1 (TAK1), therefore dephosphorylating STAT3 without affecting its total expression level [106]. The dopamine receptor D2 (DRD2)-targeting drug thioridazine inhibits TNBC cell self-renewal through reducing DRD2-mediated STAT3 activation [107]. Due to the highly conserved structures among STAT family members, targeting the upstream regulators always results in the wide-spectrum inhibition of all STAT proteins, causing off-target effects. Therefore, directly targeting STAT3 and/or inhibiting its functions may be more promising strategies for developing safe and effective anticancer therapeutics.

### Directly bind to STAT3 and inhibit its activation

Due to advances in the understanding of the structural biology of STAT3, small molecule inhibitors have been developed to directly bind to STAT3 and inhibit its activity. Currently, many small molecule inhibitors have been designed to target the SH2 domain and block its phosphorylation, dimerization, and nuclear translocation. Several STAT3-binding small molecule inhibitors that are under preclinical and clinical investigations have shown excellent efficacy in TNBC cells in vitro and in vivo.

Recently, a dual-luciferase assay-based screening of 1563 compounds for STAT3 inhibitors was performed, leading to the identification of Bt354 [108]. Further studies have shown that Bt354 inhibits STAT3 phosphorylation and nuclear translocation, which may be attributed to the binding of this compound to the SH2 domain of STAT3. Bt354 did not cause significant changes in the expression of STAT3 upstream regulators JAK2 and Src, indicating a specific targeting effect on STAT3 [108]. Moreover, this small molecule inhibitor also suppresses the viability of TNBC cells with constitutively activated STAT3, induces the G2/M phase arrest and late apoptosis, and impairs cell migration in vitro and represses the growth of TNBC xenograft tumors in vivo [108]. Additionally, several natural products, including osthole [109], arctigenin [110], and alantolactone [111] have also been shown to directly bind to the SH2 domain of STAT3, inhibit its phosphorylation and activation, and suppress the growth and metastasis of TNBC in vitro and in vivo. Cryptotanshinone is a well-documented natural product inhibitor of STAT3, which also binds to the SH2 domain and inhibits the phosphorylation and dimerization of STAT3 [112]. KYZ3, a synthetic derivative of cryptotanshinone has recently been developed and shown to exert anticancer activity in TNBC cells in vitro and in vivo through binding to and inhibiting STAT3 activation [113]. However, none of these compounds have been evaluated for their binding affinity to STAT3. Their selectivity among STAT3 and other STAT family members is yet to be determined.

### Inhibit STAT3 phosphorylation or acetylation

Except for the STAT3-binding small molecule inhibitors that we discussed above, a number of natural products and their derivatives were found to inhibit STAT3 phosphorylation and/or nuclear translocation without affecting the upstream regulators. Sesquiterpene lactones, which are enriched in the hexane fraction from *Inula helenium* L. have been shown to suppress tumor growth in vitro and in vivo by inhibiting STAT3 phosphorylation and decreasing the expression of the downstream target genes, including cyclin D1, c-Myc, and Bcl-2 [114]. Another crude extract from the fruits of *Rhus coriaria* was also discovered to inhibit angiogenesis, tumor growth and metastasis in TNBC models in vitro and in vivo by repressing STAT3 phosphorylation and STAT3-mediated VEGF expression [115]. Moreover, several natural compounds and derivatives, including schisandrin B [116], eupalinolide J [117], galiellalactone analogs 16 and 17 [118], and ursolic acid derivative FZU-03,010 [119] have shown in vitro and in vivo efficacy in TNBC models through inhibition of STAT3 phosphorylation and/or nuclear translocation. None of them have been investigated for the binding ability with STAT3. Considering that these compounds did not show any significant effects on STAT3 regulators and interactive proteins, further studies for examining the potential binding between STAT3 and these compounds would provide important information regarding their underlying molecular mechanisms.

Of note, several approved drugs have shown potent inhibitory effects on pSTAT3 and may be repositioned as anticancer drugs. Niclosamide, an FDA-approved anthelmintic drug was identified as a potent STAT3 inhibitor. A recent study demonstrated that niclosamide not only inhibits TNBC cell viability but also sensitizes TNBC cells to ionizing irradiation (IR) by blocking IR-induced STAT3 phosphorylation and activation [120]. Flubendazole, another wildly used anthelmintic agent and disulfiram, a clinical drug for treating chronic alcoholism were found to eradicate TNBC stem cells-like cells that express high levels of pSTAT3 [121, 122]. Further studies showed that both drugs were able to cause TNBC cell growth arrest and apoptosis in vitro and suppress TNBC tumor growth, angiogenesis, and metastasis in vivo by inhibiting STAT3 [121, 122]. Moreover, salinomycin, an antibacterial and coccidiostat ionophore therapeutic drug and metformin, an antidiabetic drug have exhibited potent inhibitory effects on STAT3 phosphorylation and TNBC cell growth in vitro [123, 124]. However, further evaluation of their anti-TNBC efficacy in in vivo models is critically needed.

Recent studies have disclosed that targeting STAT3 acetylation may be a potential therapeutic approach for treating cancer. SH-I-14, a newly synthesized carbazole was shown to inhibit STAT3 phosphorylation through increasing SHP-1 expression [125]. A follow-up study reported that SH-I-14 also inhibited STAT3 acetylation and disrupted DNMT1-STAT3 interaction, resulting in DNA demethylation and re-expression of tumor suppressor genes [126]. Its in vitro and in vivo activity has also been demonstrated in TNBC model, suggesting the effectiveness of inhibiting STAT3 acetylation in TNBC therapy.

### Block STAT3-DNA binding

STAT3 induces the expression of its downstream targets through binding to DNA and activating the transcription. Therefore, inhibition of STAT3-DNA binding has been considered as a promising strategy to develop targeted cancer therapies. Several STAT3-DNA binding inhibitors have been developed and shown potent anticancer efficacy in TNBC cells. Methylsulfonyl-methane (MSM), a dietary supplement was found to inhibit TNBC cell viability and induce apoptosis by blocking the DNA binding abilities of STAT3 to *VEGF* promoter and STAT5 to *IGF-1R* (IGF-1 receptor) promoter and repressing the expression of VEGF and IGF-1R [127]. Considering the extremely low toxicity of MSM, it could be developed as a preventive agent for cancers harboring overexpressed and aberrantly activated STAT3. Two natural compounds, isoharringtonine and salidroside have also been demonstrated to exert their anti-TNBC activities by blocking the binding of STAT3 to *Nanog* and *MMP2* promoters, respectively [128, 129]. However, their binding affinity to STAT3 and in vivo efficacy are yet to be studied.

As discussed above, several strategies (as shown in Fig. 3) have been developed to inhibit STAT3 signaling, i.e. 1) targeting the upstream regulators, 2) directly binding to STAT3 SH2 domain and inhibiting its activation, 3) inhibiting STAT3 phosphorylation or acetylation, and 4) blocking STAT3-DNA binding. Many small molecules have been developed and shown efficacy in preventing and treating TNBC in preclinical studies (Table 1). Several STAT3 inhibitors also enter clinical trials [130–148], which have been summarized in Table 2. There are other STAT3-targeting strategies (as shown in Fig. 3) that have not been examined, including 1) inhibiting the binding of STAT3 with its co-activators (e.g.*,* NCOA/SRC1a, APE/Ref-1, and CBP/p300) and repressing its transcriptional activity, 2) modulating the binding of STAT3 with other interactive proteins (e.g.*,* SMYD2 and TRAF6) that regulate its activity and stability, and 3) developing STAT3-targeting PROTACs (proteolysis targeting chimeras) for promoting STAT3 ubiquitination and proteasomal degradation. Because most of the small molecule STAT3 inhibitors have been developed to inhibit its phosphorylation and activation but not affect the protein stability, long-term treatment of these inhibitors may result in the compensatory activation of other signaling pathways, finally causing drug resistance. Therefore, small molecules, such as PROTACs that can induce STAT3 protein degradation may be used more efficiently in combination with current inhibitors for cancer therapy.

**Table 2:** Summary of STAT3 inhibitors in clinical trials

Inhibitors	Target	ClinicalTrials ID	Condition or disease	Phase	References
STAT3 DECOY	STAT3	NCT00696176	Head and neck cancer	Early phase 1	[130]
AZD9150 (IONIS-STAT3Rx or ISIS-STAT3Rx)	STAT3	NCT01563302	Advanced cancers, DLBCL	Phases 1 & 2	[131, 132]
		NCT02417753	Ovarian cancer, GIC	Phase 2	
		NCT01839604	HCC	Phase 1	
		NCT02983578	GIC, lung cancer, etc.	Phase 2	
		NCT03527147	NHL, DLBCL, NHL, DLBCL	Phase 1	
		NCT02549651	DLBCL	Phase 1	
		NCT03421353	Advanced solid tumors	Phases 1 & 2	
TTI-101 (C188–9)	STAT3	NCT03195699	Breast cancer, HNSCC, NSCLC, etc.	Phase 1	[133]
OPB-51602	STAT3	NCT02058017	Nasopharyngeal carcinoma	Phase 1	[134]
		NCT01867073	Advanced solid tumors	Phase 1	
		NCT01423903	Advanced cancer	Phase 1	
OPB-31121	STAT3	NCT00955812	Advanced cancer, solid tumor	Phase 1	[136]
OPB-111077	STAT3	NCT01711034	Solid tumors	Phase 1	[137]
Napabucasin (BBI608 or GB201)	STAT3	NCT03647839	MCC	Phase 2	[135]
		NCT03522649	Previously treated MCC	Phase 3	
		NCT02826161	NSCLC	Phase 3	
		NCT02993731	Pancreatic ductal carcinoma	Phase 3	
Pyrimethamine	STAT3	NCT01066663	CLL, SLL	Phases 1 & 2	[138]
		NCT03057990	Myelodysplastic syndromes	Phase 1	
Simvastatin	STAT3	NCT02390843	Retinoblastoma, clear cell sarcoma, renal cell carcinoma, rhabdoid tumor, etc.	Phase 1	[139]
DSP-0337	STAT3	NCT03416816	Neoplasms	Phase 1	[140]
Cetuximab	EGFR	NCT01445405	Squamous carcinoma, head and neck cancer, etc.	Phase 1	[141]
Lapatinib	EGFR	NCT00105950	Breast neoplasms	Phase 2	[142]
Dasatinib	c-Src	NCT02680951	AML	Phase 1	[143]
SC-43	SHP-1	NCT03443622	Refractory solid tumor	Phase 1	[144]
ASN002	JAK	NCT02440685	Lymphoma, leukemia	Phases 1 & 2	[145]
SAR302503	JAK2	NCT01420783	Hematopoietic neoplasm	Phase 2	[146]
AZD1480	JAK2	NCT01112397	Solid malignancies	Phase 1	[147]
WP1066	JAK2	NCT01904123	Metastatic melanoma, recurrent glioblastoma, etc.	Phase 1	[148]

*AML* Acute myeloid leukemia, *CLL* Chronic lymphocytic leukemia, *DLBCL* Diffuse large B-cell lymphoma, *GIC* Gastrointestinal cancer, *HCC* Hepatocellular carcinoma, *HNSCC* Head and neck squamous cell carcinoma, *MCC* Metastatic colorectal cancer, *NHL* Non-Hodgkin lymphoma, *NSCLC* Non-small cell lung cancer, *SLL* Small lymphocytic leukemia

## Conclusions

TNBC is still a treatable but incurable disease with complex genetic heterogeneity. The STAT3 oncogene is overexpressed and constitutively activated in TNBC and is associated with the high metastatic risk and poor survival outcomes. Moreover, STAT3 not only acts as a transcription factor to activate the expression of its downstream target genes but also localizes to mitochondria and regulates its functions, then regulating the various aspects of TNBC cells. Many STAT3-targeted therapies have been successfully developed and shown efficacy in preclinical models of TNBC in vitro and in vivo; several STAT3 inhibitors even enter clinical trials and are currently under investigation in various human cancers, including TNBC. In addition to its role in cancer cells, STAT3 also plays a pivotal role in the immune system. Indeed, STAT3 inhibitors have been found to suppress tumor cells but also boost immune cell responses. Therefore, the STAT3 oncogene is a promising target for TNBC prevention and therapy.

Of note, targeting STAT3 alone has shown excellent anti-TNBC activities in preclinical settings. However, TNBC has been reported to harbor multiple genetic alterations, including STAT3 overexpression and constitutive activation which contribute to the initiation, progression, metastasis, and drug resistance of this disease. Therefore, STAT3 inhibition combined with other targeted therapies may be more effective in treating TNBC. Considering that STAT3 plays a crucial role in chemoresistance, the combination of STAT3 inhibitors with other chemotherapies may exert synergistic effects in treating TNBC. Therefore, further studies are warranted to demonstrate the preventive and therapeutic efficacy of STAT3 inhibitors alone or in combination with chemotherapy and/or other targeted therapies in clinical studies. Moreover, new targeting strategies, i.e. inducing the degradation of STAT3 protein through PROTAC or inhibiting the binding of STAT3 to its co-activators and other interactive proteins can be examined, which may lead to more specific and effective inhibitors for TNBC prevention and therapy.

## Abbreviations

ABC: ATP-binding cassette
AML: Acute myeloid leukemia
APE/Ref-1: Apurinic/apyrimidinic endonuclease-1/redox factor-1
ATO: Arsenic trioxide
Bax: Bcl-2-associated x protein
Bcl-2: B-cell lymphoma-2
Bcl-xL: B-cell lymphoma-extra Large
bFGF: Basic fibroblast growth factor
CBP: CREB-binding protein
CCD: Coiled-coil domain
CLL: Chronic lymphocytic leukemia
DBD: DNA binding domain
DLBCL: Diffuse large B-cell lymphoma
DRD2: Dopamine receptor D2
EGF: Epidermal growth factor
EGFR: Epidermal growth factor receptor
ER: Estrogen receptor
EZH2: Enhancer of zeste homolog 2
FAK: Focal adhesion kinase
FGFR: Fibroblast growth factor receptor
GGNBP2: Gametogenetin-binding protein 2
GIC: Gastrointestinal cancer
gp130: Glycoprotein 130
GPER: G protein-coupled estrogen receptor
GPER-1: G protein-coupled estrogen receptor-1
GRIM-19: Gene associated with retinoic-interferon-induced mortality 19
HCC: Hepatocellular carcinoma
HER2: Human epidermal growth factor receptor 2
HGF: Hepatocyte growth factor
HIF-1α: Hypoxia-inducible factor 1-alpha
HN1L: Hematological and neurological expressed 1-like
HNSCC: Head and neck squamous cell carcinoma
HSP90: Heat shock protein 90
IGF-1R: IGF-1 receptor
IGFR: Insulin-like growth factor receptor
IL-10R: Interleukin-10 receptor
IL-22: Interleukin-22
IL-6: Interleukin-6
IL-6R: Interleukin-6 receptor
IR: Ionizing irradiation
JAKs: Janus kinases
LECs: Lymphatic endothelial cells
LEPRb: Long form of leptin receptor
LVs: Lymphatic vessels
MCC: Metastatic colorectal cancer
miRNA: MicroRNA
MMP: Matrix metalloproteinase
MRP2: Multidrug resistance protein 2
MSK1: Mitogen- and stress-activated protein kinase-1
MSM: Methylsulfonyl-methane
NF-κB: Nuclear factor-kappa B
NHL: Non-Hodgkin lymphoma
NSCLC: Non-small cell lung cancer
Oct-4: Octamer-binding transcription factor-4
PARP: Poly (ADP-ribose) polymerase
pATF2: Phosphorylated activating transcription factor 2
pc-Jun: Phosphorylated c-Jun
PD-L1: Programmed death ligand 1
PGG: 1,2,3,4,6-penta-O-galloyl-beta-D-glucose
PIAS: Protein inhibitor of activated STAT
PR: Progesterone receptor
PROTACs: Proteolysis targeting chimeras
pSTAT3: Phosphorylated STAT3
PTPRT: Protein tyrosine phosphatase receptor T
PTPs: Protein tyrosine phosphatases
ROS: Reactive oxygen species
SH2: SRC homology 2
SHP-1/2: Src homology domain-containing tyrosine phosphatases 1/2
SLL: Small lymphocytic leukemia
SMYD2: SET and MYND domain 2
SOCS: Suppressors of cytokine signaling
Sox2: SRY-related HMG-box 2
STAT3: Signal transducer and activator of transcription 3
TAD: Transactivation domain
TAK1: Transforming growth factor β-activated kinase 1
TC-PTP: T-cell protein-tyrosine phosphatase
tDRs: Transfer RNA-derived fragments
TNBC: Triple negative breast cancer
TNFRSF1A: Tumor necrosis factor receptor superfamily member 1A
TRAF6: Tumor necrosis factor receptor-associated factor 6
UTR: 3′-untranslated region
VEGF: Vascular endothelial growth factor
Wwox: WW domain-containing oxidoreductase
XPO1: Exportin 1
